# Investigating the Effectiveness and Acceptability of Oral Health and Related Health Behaviour Interventions in Adults with Severe and Multiple Disadvantage: Protocol for a Mixed-Methods Systematic Review

**DOI:** 10.3390/ijerph182111554

**Published:** 2021-11-03

**Authors:** Laura J. McGowan, Emma C. Joyes, Emma A. Adams, Aishah Coyte, Richard Gavin, Catherine Richmond, Hosein Shabaninejad, Fiona Beyer, Angela Broadbridge, Kevin Dobson, David Landes, Suzanne Moffatt, Richard G. Watt, Falko F. Sniehotta, Ruth Freeman, Martha Paisi, Clare Bambra, Dawn Craig, Eileen Kaner, Sheena E. Ramsay

**Affiliations:** 1 Population Health Sciences Institute, Newcastle University, Newcastle upon Tyne NE2 4AX, UK; emma.joyes@newcastle.ac.uk (E.C.J.); emma.adams@newcastle.ac.uk (E.A.A.); aishah.coyte@newcastle.ac.uk (A.C.); catherine.richmond@newcastle.ac.uk (C.R.); Hosein.Shabaninejad@newcastle.ac.uk (H.S.); fiona.beyer@newcastle.ac.uk (F.B.); suzanne.moffatt@newcastle.ac.uk (S.M.); falko.sniehotta@newcastle.ac.uk (F.F.S.); Clare.bambra@newcastle.ac.uk (C.B.); dawn.craig@newcastle.ac.uk (D.C.); Eileen.kaner@newcastle.ac.uk (E.K.); sheena.ramsay@newcastle.ac.uk (S.E.R.); 2 Northumbria Healthcare, NHS Foundation Trust, Newcastle upon Tyne NE27 0QG, UK; richard.gavin2@nhs.net; 3 Fulfilling Lives Newcastle/Gateshead, Gateshead NE8 4DY, UK; Angela.Broadbridge@fulfillinglives-ng.org.uk (A.B.); kevin.dobson@crisis.org.uk (K.D.); 4 Public Health England, London SE1 8UG, UK; David.Landes@phe.gov.uk; 5 Department of Epidemiology and Public Health, University College London, London WC1E 7HB, UK; r.watt@ucl.ac.uk; 6 Dental Health Services Research Unit, University of Dundee, Dundee DD1 4HN, UK; r.e.freeman@dundee.ac.uk; 7 School of Nursing and Midwifery, University of Plymouth, Plymouth PL4 8AA, UK; martha.paisi@plymouth.ac.uk

**Keywords:** multiple disadvantage, homelessness, repeat offending, substance misuse, oral health, smoking, diet, systematic review, evidence synthesis, health inequalities

## Abstract

Increasing numbers of people in England experience homelessness, substance use, and repeated offending (known as ‘severe and multiple disadvantage’; SMD). Populations experiencing SMD often have extremely poor oral health, which is closely inter-linked with high levels of substance use, smoking, and poor diet. This study aims to undertake an evidence synthesis to identify the effectiveness, resource requirements, and factors influencing the implementation and acceptability of oral health and related health behaviour interventions in adults experiencing SMD. Two systematic reviews will be conducted using mixed-methods. Review 1 will investigate the effectiveness and resource implications of oral health and related health behaviours (substance use, smoking, diet) interventions; Review 2 will investigate factors influencing the implementation of such interventions. The population includes adults (≥18 years) experiencing SMD. Standard review methods in terms of searches, screening, data extraction, and quality appraisal will be conducted. Narrative syntheses will be conducted. If feasible, a meta-analysis will be conducted for Review 1 and a thematic synthesis for Review 2. Evidence from the two reviews will then be synthesised together. Input from people with experience of SMD will be sought throughout to inform the reviews. An initial logic model will be iteratively refined during the review.

## 1. Introduction

The overlap between people experiencing homelessness, problematic substance use and involvement with the criminal justice system is substantial and these are key indicators of severe and multiple disadvantage (SMD) [1]. Over two-thirds of people experiencing homelessness report problematic substance use or involvement with the criminal justice system; similarly 63% of offenders and 40% of those experiencing problematic substance use experience the other two issues [1]. In England over 250,000 people each year are involved with at least two out of the three issues; with 58,000 experiencing all three [1]. Although there is some evidence of a decrease in the number of people sleeping rough over the past two years [2], overall there has been large increases in the numbers of individuals experiencing SMD over the past decade; e.g., rough sleeping increased by 132% in England since 2010 [3]. SMD is associated with persistent, low-level offending and short prison or community sentences, resulting in disrupted lifestyles which are often interlinked with substance misuse (dependence on drugs/alcohol) [1,4]. The consequences of SMD have major adverse health impacts on affected individuals [1,4,5]. SMD populations have very high levels of mental and physical ill-health [3,5].

Poor oral health is one of the three most common physical health problems faced by SMD populations [5]. SMD groups have disproportionately high levels of tooth loss, untreated dental disease (caries/tooth decay, periodontal disease), infections, and pain [6,7,8]. Peer-led research showed that 90% out of 262 SMD individuals interviewed had issues with their mouth, 60% experienced dental pain, and 70% had lost teeth since becoming homeless [9].

Oral health has an integral and bi-directional link with common health behaviours in SMD groups, particularly smoking, high sugar consumption, drug use, and excessive alcohol intake (high strength alcohol, e.g., spirits). These constitute key risk factors for physical and mental ill-health, and oral diseases, including dental caries (decay), tooth erosion, periodontal disease, tooth loss and oral cancers [10].

SMD groups also suffer from the most severe complications of dental diseases compared with the general population, including dental or orofacial pain, oral cancer and abscesses or infections [11]. These complications are often untreated or result in emergency treatment at dental emergency clinics where available, or Accident & Emergency departments [12]. Access to routine and preventive healthcare is extremely poor and non-attendance is common; poor attendance is often a result of the lived experience of homelessness, including disrupted/chaotic lifestyles, lack of knowledge, anxiety and social isolation [13,14]. Further, characteristics of the healthcare system (e.g., lack of training in dealing with socially excluded groups, the cost of care, and problems in registering patients with no fixed address) also serve as prominent barriers to dental care for SMD groups [14]. Due to these barriers to accessing care, people facing SMD often resort to increased drug and alcohol to manage issues such as dental pain, creating a cycle of continuous deterioration of their oral health and perpetuating the cycle of SMD [6,7,13,15].

**Figure 1:**
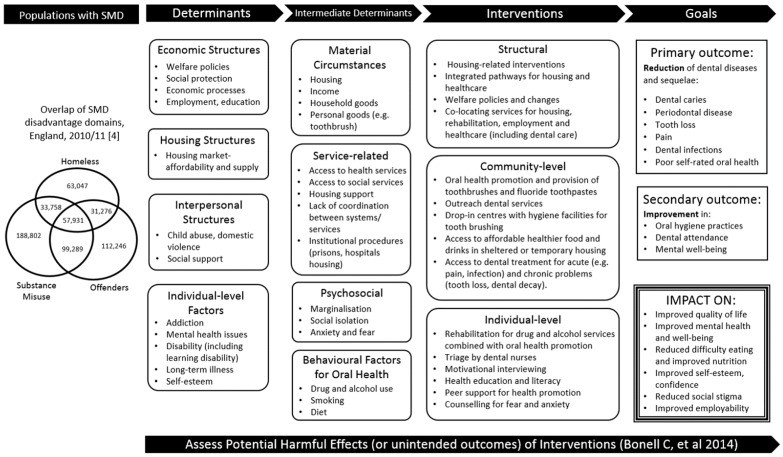
Initial logic model laying out the theoretical framework for identifying interventions to improve the oral health and related behaviours of adults with severe and multiple disadvantage (SMD).

Whilst these problems of poor oral health and related health behaviours in SMD groups have been well described, limited information for policymakers and service providers exists on how to effectively address oral health needs of SMD groups and thereby reduce health inequalities. To address this gap, we aim to undertake an evidence synthesis to identify interventions that are effective, acceptable and sustainable in improving the oral health and related health behaviours of adults experiencing SMD. Two complementary systematic reviews will be conducted using a mixed-methods approach. In mixed-methods syntheses, quantitative and qualitative studies focused on the same topic are reviewed and brought together in order to generate a broader range of evidence to inform decision-making [16] and improve interventions and their implementation [17]. Review 1 will synthesise quantitative comparative trials to review the effectiveness of interventions in improving oral health and related health behaviours (smoking, drug and alcohol misuse, and diet) in adults with SMD, and the resource implications, including associated costs, of these interventions. Review 2 will synthesise qualitative evidence to investigate factors that influence implementation of these interventions, including settings, acceptability, and potential adverse effects of interventions. Quantitative outcomes relating to implementation (e.g., numeric ratings of acceptability) will also be considered for Review 2. Table 1 shows an overview of the two complementary reviews. The reviews will be underpinned by the development of an initial logic model (see Figure 1) to capture the conceptual framework of interventions identified, and input from people with lived experience of SMD.

## 2. Materials and Methods

This protocol follows the Preferred Reporting Items for Systematic Reviews and Meta-Analyses-Protocol (PRISMA-P) guidelines [18] (PRISMA-P checklist is included in Supplementary Material S1). The review is registered with PROSPERO (CRD42020202416) [19]. Future changes to the protocol will be clearly stated in PROSPERO.

Our reviews will be underpinned by our initial logic model (Figure 1). This draws on initial scoping of literature, input from people who have experience of SMD on determinants of oral health, and reflects the collective knowledge of our team (expertise in multiple exclusion, public health, inequalities, evidence synthesis, health psychology, dental public health, complex interventions). We will refine this logic model to create a conceptual framework to underpin effective interventions [20,21]. The logic model will provide a deeper understanding of why and in which circumstances interventions are successful (or not). The framework takes a whole-systems approach to unpack determinants linking SMD with oral health and health behaviours. We will use our initial logic model to help define the scope of the reviews, and will continue to use it to conceptualise and guide our reviews [22]. We will refine the logic model through comparison with emerging findings from the reviews.

‘Experts by Experience’ (individuals with lived experience of SMD) will have input into all stages of the review including its scope, emerging findings and interpretation via regular discussions with our partners from Fulfilling Lives Newcastle/Gateshead (an organisation that supports individuals experiencing SMD). These insights will ensure that the reviews are meaningful and relevant for the target population [23,24,25].

### 2.1. Eligibility Criteria for Reviews

To identify relevant evidence for the review, a clear definition of the eligible study participants, interventions, comparators, outcomes and study designs of interest are required. These are described below.

#### 2.1.1. Population and Setting

Adults (≥18 years) with SMD comprising homelessness (rough sleeping and other forms of highly insecure/inadequate accommodation including but not limited to night shelters, hostels, living temporarily with friends/family, living in unfit housing, and living under the threat of eviction [3,26]); repeat offending (persistent, low-level offending and short prison or community sentences [27]); or problematic substance use (use of drugs or alcohol in a harmful way that has negative effects on health [28]) where this co-occurs with homelessness and/or repeat offending [1].

#### 2.1.2. Interventions

Interventions will include those at structural, community and individual levels, in line with frameworks that have been used in previous reviews on health inequalities [29]. Examples from our scoping searches include: structural-level interventions related to housing, such as Housing First England (a housing and support programme for people who are experiencing homelessness [30]), co-locating services for housing, rehabilitation, employment and healthcare; community-level interventions including oral health promotion in temporary housing; and individual-level interventions such as, rehabilitation services, motivational interviewing, peer-support.

#### 2.1.3. Comparators

For Review 1 (effectiveness/resource implications), studies that evaluate an eligible intervention against any comparator intervention, e.g., standard care (or no current care provision), will be eligible. Studies with no comparator (i.e., single arm studies) will not be eligible for inclusion for Review 1, however will be eligible for Review 2 (implementation).

#### 2.1.4. Outcomes

Review 1 (effectiveness/resource implications): Primary outcomes include oral health (e.g., dental caries, periodontal disease, tooth loss, pain, dental infections), health behaviours (poor diet, smoking, drug and alcohol misuse/dependence), economic outcomes (cost-effectiveness, costs, resource use), and adverse effects. Secondary outcomes include social and economic outcomes such as mental wellbeing, health-related quality of life, self-esteem, employment, and income. Studies must report at least one of the primary outcomes to be eligible for inclusion.

Review 2 (implementation): For the implementation review, outcomes include views of SMD groups and other stakeholders on acceptability of interventions, including content, settings, factors related to uptake and maintenance, perceived impact, and potential harmful consequences of interventions. We will also explore quantitative outcomes relating to implementation and acceptability (e.g., retention).

#### 2.1.5. Study Design

For Review 1, all comparative study designs will be eligible, including: Individual and cluster randomised controlled trials (RCTs), quasi-experimental studies, prospective, and comparative non-RCTs (e.g., cohort studies and case control studies). In addition to measures of effectiveness, any resource or cost data within these effectiveness studies will be captured. To complement this, comparative economic evaluations reporting costs of interventions will also be eligible for inclusion, including cost-minimization analysis (CMA), cost-effectiveness analysis (CEA), cost–benefit analysis (CBA), cost–utility analysis (CUA), model-based economic evaluation, and interrupted time series (ITS).

For Review 2 (implementation), eligible studies will include both qualitative studies (e.g., interviews, focus groups, ethnographies), and quantitative studies (e.g., questionnaires, surveys), and mixed-methods studies.

Results from pilot/feasibility or single-arm studies will not be eligible for inclusion in Review 1, however will be included in Review 2 if containing outcomes related to implementation. Systematic and non-systematic reviews, individual case reports, commentaries, editorials, letters, and opinion pieces will be ineligible for either review. Any studies published as abstracts or conference presentations will be eligible for inclusion, provided that any outcome data of interest are sufficiently reported.

#### 2.1.6. Limits

No limit on date, country or language will be applied. All attempts will be made to translate papers, but where this is not possible we will include the study details of such papers as an appendix for completeness.

### 2.2. Information Sources and Search Strategy

The search strategy was developed in collaboration with an information specialist in our review team. The strategy was initially developed in MEDLINE. A combination of keyword and subject heading terms were used, which were then developed in conjunction with the project team, including clinical experts, as well as by referring to previously identified papers. The final strategy was then verified against relevant articles identified during initial scoping. The search strategy was developed in MEDLINE, Ovid (1946 to 27 July 2020) and translated on 29 July 2020 to the following databases: EMBASE, Ovid (1974 to 27 July 2020); CINAHL, Ebsco (1981–present); APA PsycINFO (1806 to July Week 3 2020); and Scopus. The MEDLINE search is available within our PROSPERO registration (CRD42020202416) [19] and included as Supplementary Material S2. Search results will be downloaded and loaded into bibliographic software (Endnote) and deduplicated. We will conduct forward and backward citation searching on all included studies following eligibility screening. A grey literature search will also be conducted, including Google Incognito and relevant charity and organisation websites (e.g., Fulfilling Lives, Crisis).

### 2.3. Data Collection

#### 2.3.1. Data Management

All references generated from the search will be uploaded from Endnote and managed in Covidence, an online screening and data extraction tool for systematic reviews [31].

#### 2.3.2. Study Selection

Screening and searching for the reviews (effectiveness and implementation) will be carried out simultaneously to maximise efficiency. The process for identifying studies for review will follow the stages of the Preferred Reporting Items for Systematic Reviews and Meta-Analyses (PRISMA) statement [32]. The number of records identified and included/excluded at each stage of the screening process will be reported in a PRISMA flow diagram in our final publication of results.

All titles and abstracts identified in the searches will be screened for eligibility based on the agreed inclusion criteria. This will be conducted independently by two reviewers. Initially, inclusion/exclusion criteria will be piloted on a selection of studies and the reviewers will discuss agreements and disagreements on screening. Clarifications on eligibility criteria will be documented.

The title and abstract screening will result in a set of potentially relevant records, which will be assessed further at full-text review. Once full-text publications are obtained, these will be further screened independently by two reviewers for relevance based on the eligibility criteria. Any discrepancies will be resolved by consulting a third independent reviewer to reach consensus.

#### 2.3.3. Data Extraction

Data extraction will be conducted by one reviewer and verified by a second reviewer for all included studies. Any discrepancies will be resolved by a third reviewer. Standardised data extraction forms will be developed and piloted in Excel for the different aspects of the review (effectiveness, costs/resource implications, and implementation) [22,33]. Regarding data extraction for the economic evaluation, dummy tables will be developed to include cost values and resource use based upon related conceptual paper(s) and discussion within the team.

For both reviews, the following types of study information will be extracted:

- Design, setting, country, aims, date, inclusion/exclusion criteria, population characteristics (homeless; substance users; repeat offenders; multiple disadvantage), sample characteristics (e.g., age/ethnicity, duration of SMD characteristic), sample size, type of analysis, intervention details (based on the template for intervention description and replication [TIDieR] [34]); outcomes (definition, unit of measurement, number of participants included in analysis, size of effect (for dichotomous outcomes—absolute and relative risks (or odds ratios) and risk (or rate) differences (unadjusted/adjusted); for continuous outcomes—the mean change and measure of variance from baseline (or at both baseline and final visit), or mean difference between treatments (unadjusted/adjusted); for time-to-event analysis—the number of events in each arm, median time to event and a hazard ratio and *p*-value (unadjusted/adjusted)), measure of precision for each effect estimate (95% confidence intervals, standard error or standard deviation), cost values/resource use data related to interventions and outcomes (within effectiveness studies); factors affecting acceptability; barriers and facilitators; perceived benefits/harms (for qualitative studies ‘findings’ or ‘results’ will be considered as data; outcomes on these findings, if reported in any quantitative studies will also be included)

For comparative economic evaluation studies, the following data will be extracted:

- Perspective of study (and if this relates to costs being evaluated); time horizon(s) over which costs and consequences are being evaluated; dates of the estimated resource, quantities and unit costs; Choice of Discount rate(s) used for costs and outcomes; approaches and data source used to estimate resource use for model-based economic evaluation studies; cost-effectiveness ratios

#### 2.3.4. Quality Assessment

Two reviewers will independently appraise the studies meeting the inclusion criteria to assess the risk of bias. RCTs will be assessed using domain-based evaluation recommended by Cochrane review methodology [33]. Non-randomised studies will be appraised using established tools such as ROBINS-I [22,33,35]. The quality of studies included in the qualitative review will be assessed using established tools such as, Critical Appraisal Skills Program (CASP) tool [36]. Any economic evaluations will be assessed using the Drummond checklist [37]. The results of the quality assessment will be summarised in a table and provided with the published results. Studies will not be excluded on the basis of low quality; quality assessment will be used primarily to aid interpretation of analysis and may inform sensitivity analyses.

### 2.4. Data Synthesis

Evidence from the two reviews will be synthesised using a results-based convergent design, whereby separate analyses will be conducted for each review using separate synthesis methods, and the results of each will be brought together in a final stage of synthesis [38,39].

For Review 1, evidence will be synthesised for each group of outcomes (e.g., oral health, individual health behaviours). If meta-analysis is not feasible, a narrative synthesis of findings will be conducted and reported in line with the Synthesis Without Meta-analysis (SWiM) guidelines [40]. Where feasible, appropriate meta-analytic methods will be used: Mantel-Haenszel for odds ratios from dichotomous data, weighted mean difference (or standardised weighted mean difference if different metrics are used) for continuous outcomes, generic inverse variance method for time to event data [33,41]. Heterogeneity between studies will be assessed by visual inspection of plots of the data, from the chi-square test for heterogeneity, and the I^2^ statistic. Possible reasons for heterogeneity will be explored where feasible. Publication bias in the reported studies will be investigated using a funnel plot. Results will be reported using PRISMA-E guidelines for equity-focused systematic reviews [42].

We will categorise studies according to duration of follow-up; this will depend on the studies and will be outcome-dependent, but is likely to include short, medium, and longer term. We will then assess whether there is any variation over time by carrying out sub-group analysis (if statistical pooling is possible) or narratively comparing effectiveness of studies in the three categories. This will help assess the effectiveness of interventions over time, which is important in terms of identifying sustainable interventions. We will conduct sub-group analysis by populations in specific domains of multiple disadvantage (i.e., homelessness, offenders), as well as two or more domains of SMD, where feasible. A previous review of health interventions in people with multiple exclusion suggests a difference in effectiveness by gender and age [43].We will explore the feasibility to undertake sub-group analysis by age (younger vs. older adults), gender, use of theory in intervention development, and outcome type (e.g., oral health, smoking).

A narrative synthesis will be undertaken for the costs related component of Review 1 to describe the similarities and differences in study questions, methods and results. Cost estimates will be adjusted to the target currency and price year using The Campbell and Cochrane Economics Methods Group (CCEMG) and the Evidence for Policy and Practice Information and Coordinating Centre (EPPI-Centre)’s web-based cost converter [44]. Should it be feasible, the adjusted cost values (unit cost for each items of interventions) will also be provided using the Personal Social Services Research Unit (PSSRU)’s unit costs of health and social care [45]. Given the nature of the interventions likely to emerge from the review and the diversity of outcomes likely to impact on well-being and quality of life, a cost-consequence analysis (CCA), presenting costs (e.g., intervention implementation costs) alongside consequences, would be undertaken.

For Review 2 a narrative synthesis will be undertaken. A major focus is to identify factors affecting implementation and sustainability of interventions. Our review will focus on factors, such as the relevance of settings, acceptability (to service users and providers) and potential adverse effects of interventions [21,46]. A thematic synthesis will be conducted to code, analyse and synthesise studies [47,48]. Recurring themes will be explored and analysed to enable conclusions to be drawn. Evidence synthesis will be presented narratively and in a synthesis table and reported following ENTREQ guidelines [49].

In line with a results-based convergent approach [38], evidence from the two reviews will be scrutinised together, so that inferences based on close examination of both qualitative and quantitative data together can be made. The logic model will be developed and refined throughout, and results will be mapped to the model accordingly.

## 3. Discussion

Improving the oral health and related health behaviours of populations facing SMD is an important yet under-researched health-inequalities issue. A systematic review of evidence is needed to identify and investigate interventions that are effective in improving the oral health and related health behaviours of SMD populations. There is also a need to understand factors influencing the implementation of these interventions to ensure that interventions are sustainable and acceptable to SMD populations, service providers and decision makers. This review will address these evidence gaps and provide recommendations for developing interventions that have important and far-reaching implications for the health of SMD populations, with strong translational value.

The review aims to integrate the literature relating to interventions for a population experiencing social exclusion, one that is often hidden or not successfully reached by existing services. Whilst there is a developing understanding of the impact of the interrelated nature of multiple disadvantage relating to the experience of homelessness, problematic substance use, and repeat offending, a siloed approach to working with such groups is often adopted [1]. As a consequence, services and research may cater to the SMD population, but do not capture the multiplicity of their disadvantage, leading to an underreporting of such issues and the continuation of unmet needs. Such issues are reflected within the literature, for example, within research the primary aim or recruitment strategy often focuses upon one aspect of disadvantage, for example homelessness, and does not capture other disadvantage experienced. For these reasons, the search strategy coupled with the screening strategy has been carefully developed to reflect such issues. For example, the term ‘Severe and Multiple Disadvantage’ is included within the search strategy to capture the developing literature within this area. However, importantly other key terms that represent a more targeted approach to exploring disadvantage are also included, such as homelessness, substance misuse, and repeat offenders. The screening strategy has also been developed in a way that is sensitive to the under-reported nature of SMD. For example, the search strategy utilises the Boolean operator ‘OR’ for our population search and not ‘AND’, so that a broader set of literature is captured. The screening strategy then investigates the literature further for possible multiplicity of disadvantage.

Though careful consideration was taken in the development of our search strategy, there is the possibility that relevant studies may be missed, particularly when bespoke terminology around our population and outcomes have been used. Due to the broad population and range of outcomes explored within this review, the team decided to adopt broad headings (or higher order headings) and search terms, such as ‘oral health’ instead of more clinical language, such as periodontal disease which is often associated within this population due to high alcohol use, consumption of sugary foods, and a lack of regular brushing [9]. Therefore, the authors recognise such an approach as a potential limitation of the review. However, this limitation will be mitigated through use of citation chaining, whereby both citations and reference lists of included studies are searched for any relevant studies not picked up in the database searches.

Further, due to the range of health outcomes under investigation and settings and contexts in which interventions may take place, the review will likely have large heterogeneity. To account for this, sub-group analyses and meta-regression will be performed where appropriate.

A strength of this review is the use of robust systematic review methods and a comprehensive search strategy. Another key strength is that insights of key stakeholders are sought, for example, those with experience of SMD as well as practitioners, so that acceptability and sustainability issues relating to interventions can be understood alongside effectiveness evidence. Such an approach aims to close the translational gap between research and practice in this area and provide a strong basis for recommendations for policy and practice.

## 4. Conclusions

Populations experiencing SMD often have extremely poor oral health, which is closely inter-linked with high levels of substance use, smoking, and poor diet. However, the evidence-base for interventions that can improve these health outcomes in SMD groups is limited. This study will provide comprehensive evidence for interventions that are effective and acceptable for improving oral health and related health behaviours in populations experiencing SMD, and identify key evidence gaps. The findings will inform further refinement, implementation and evaluation of these interventions in order to improve the health and wellbeing of SMD populations.

## Figures and Tables

**Table 1:** Overview of the two reviews.

	Review 1	Review 2
Research Question	How effective are interventions in improving oral health and related health behaviours in adults with SMD?	What factors influence implementation of interventions to improve oral health and related health behaviours in adults with SMD?
Evidence Type	Quantitative—comparative trials of interventions	Qualitative—e.g., interview/focus group studies of perceptions/experiences of interventions Quantitative evidence on implementation (e.g., retention levels, numeric ratings of acceptability)
Scope	Weighing body of evidence of trials testing effectiveness of oral health and related health behaviour interventions Focus on effect sizes, and resource implications (e.g., costs) where available Meta-analysis (if data permits)	Synthesis of stakeholders’ perceptions and experiences of interventions Focus on implementation issues, including settings, acceptability, and potential adverse effects of interventions Meta-synthesis of qualitative studies

## Supplementary Materials

The following are available online at https://www.mdpi.com/article/10.3390/ijerph182111554/s1: Supplementary Material S1: PRISMA-P checklist and Supplementary Material S2: Medline search.
