# Supplementary material for: Investigating the Effectiveness and Acceptability of Oral Health and Related Health Behaviour Interventions in Adults with Severe and Multiple Disadvantage: Protocol for a Mixed-Methods Systematic Review
Source: Int J Environ Res Public Health. 2021 Nov 3;18(21):11554. doi: 10.3390/ijerph182111554 (PMC8582803; doi:10.3390/ijerph182111554)
Supplement: Supplementary file 1 [file ijerph-18-11554-s001.zip › Supplementary Material S2_Medline v1.0 29th July 2020.pdf]

Database(s): **Ovid MEDLINE(R) and Epub Ahead of Print, In-Process & Other Non-Indexed Citations, Daily and Versions(R)** 1946 to July 27, 2020
Search Strategy:

| # | Searches | Results |
|---|---|---|
| 1 | Oral Hygiene/ | 12877 |
| 2 | Mouth Rehabilitation/ | 1441 |
| 3 | Oral Health/ | 16531 |
| 4 | exp Dental Health Services/ | 38102 |
| 5 | ((dental or oral or tooth or teeth or mouth) adj3 (health or care or hygiene or rehabilitation)).ti,ab,kw. | 60600 |
| 6 | or/1-5 | 93064 |
| 7 | Smoking/ | 139967 |
| 8 | (smoking or cigarette* or tobacco).ti,ab,kw. | 301651 |
| 9 | exp "Tobacco Use"/ | 4637 |
| 10 | Alcohol Drinking/ | 66976 |
| 11 | Alcoholism/ | 75008 |
| 12 | Alcoholics/ | 846 |
| 13 | (alcoholic* adj3 (person or people or adult or parent* or family)).ti,ab,kw. | 891 |
| 14 | "street drink*".ti,ab,kw. | 8 |
| 15 | exp Substance-Related Disorders/ | 278311 |
| 16 | exp Drug users/ | 3151 |
| 17 | Behavior, Addictive/ | 9847 |
| 18 | ((alcohol or drug* or substance*) adj2 (misuse* or abuse* or use* or addict* or dependenc* or issue* or problem*)).ti,ab,kw. | 270673 |
| 19 | (drug adj1 (habit or tak* or hard or illicit or inject*)).ti,ab,kw. | 22884 |
| 20 | or/7-19 | 772910 |
| 21 | ((sugar or sucrose or fructose or glucose) adj2 (intake or consum*)).ti,ab,kw. | 13709 |
| 22 | diet*.ti,ab,kw. | 570917 |
| 23 | "sugary food*".ti,ab,kw. | 232 |
| 24 | exp Dietary Sugars/ or Diet/ | 163303 |
| 25 | ((processed or acidic) adj1 food*).ti,ab,kw. | 3684 |
| 26 | ((sugary or fizzy or carbonated or soft) adj1 drink*).ti,ab,kw. | 4468 |
| 27 | carbonated beverages/ or sugar-sweetened beverages/ | 2994 |
| 28 | soda.ti,ab,kw. | 4176 |
| 29 | or/21-28 | 638090 |
| 30 | (severe and multiple disadvantage*).ti,ab,kw. | 4 |
| 31 | Homeless Persons/ | 7743 |
| 32 | homeless*.ti,ab,kw. | 10756 |
| 33 | ((hous* or home* or accommodat* or shelter) adj3 (insecur* or instability or unstable or stability)).ti,ab,kw. | 2860 |
| 34 | or/30-33 | 14901 |
| 35 | (probationer* or parolee* or ((repeat* or ex or re or revolving door or habitual or multiple or former* or previously*) adj1 (offen* or convict* or prisoner* or imprison* or incarcerat* or criminal*)) or (former adj3 inmate*) or ((community or probation* or parole* or reintegrat*) adj4 (prison* or offender* or criminal* or convict* or inmate*)) or ((individuals or men or women) adj2 (probation or parole)) or ((reintegrate* or reent* or return*) adj3 community)).ti,ab,kw. | 3938 |
| 36 | "criminal justice".ti,ab,kw. | 4331 |
| 37 | or/35-36 | 7796 |
| 38 | ((program* or policy or policies or strateg* or scheme* or project* or initiative* or "care package" or training or educat* or pilot or guidance or guideline* or study or pathway or treatment* or promot* or management or "support group" or process* or trial* or intervention*) adj5 (evaluat* or effect* or measur* or assess* or experiment* or impact* or feasab* or acceptab* or efficacy or perception* or belief* or uptake or consequence* or attitud* or barrier* or facilit* or motivat* or experience* or implement* or adher* or retention or retain* or reduc* or increas* or improv* or outcome* or cost* or benefit* or interview* or qualitative or ethnograph* or "focus group*")).ti,ab,kw. | 4389024 |
| 39 | "housing first".ti,ab,kw. | 299 |
| 40 | (outcome* adj5 evaluat*).ti,ab,kw. | 82642 |
| 41 | or/38-40 | 4421408 |
| 42 | (34 or 37) and (6 or 20 or 29) | 8338 |
| 43 | 41 and 42 | 3961 |

1. Ecological momentary **assessment** of daily **drug use** and harm **reduction** service utilization among **people** who **inject drugs** in non-urban areas: A concurrent mixed-method feasibility **study**.

   Biello K; Salhaney P; Valente PK; Childs E; Olson J; Earlywine JJ; Marshall BD; R Bazzi A.

   *Drug & Alcohol Dependence. 214:108167, 2020 Jul 10.*

   *[Journal Article]*

   **UI:** 32679521

**Authors Full Name**

Biello, Katie; Salhaney, Peter; Valente, Pablo K; Childs, Ellen; Olson, Jennifer; Earlywine, Joel J; Marshall, Brandon Dl; R Bazzi, Angela.

Cite
